# Fusogenic Pairings of Vesicle-Associated Membrane Proteins (VAMPs) and Plasma Membrane t-SNAREs – VAMP5 as the Exception

**DOI:** 10.1371/journal.pone.0014238

**Published:** 2010-12-06

**Authors:** Nazarul Hasan, Deborah Corbin, Chuan Hu

**Affiliations:** Department of Biochemistry and Molecular Biology, University of Louisville School of Medicine, Louisville, Kentucky, United States of America; The University of Queensland, Australia

## Abstract

**Background:**

Intracellular vesicle fusion is mediated by the interactions of SNARE (soluble *N*-ethylmaleimide-sensitive factor attachment protein receptor) proteins on vesicles (v-SNAREs) and on target membranes (t-SNAREs). The vesicle-associated membrane proteins (VAMPs) are v-SNAREs that reside in various post-Golgi vesicular compartments. To fully understand the specific role of each VAMP in vesicle trafficking, it is important to determine if VAMPs have differential membrane fusion activities.

**Methodology/Principal Findings:**

In this study, we developed a cell fusion assay that quantifies SNARE-mediated membrane fusion events by activated expression of β-galactosidase, and examined fusogenic pairings between the seven VAMPs, *i.e.*, VAMPs 1, 2, 3, 4, 5, 7 and 8, and two plasma membrane t-SNARE complexes, syntaxin1/SNAP-25 and syntaxin4/SNAP-25. VAMPs 1, 2, 3, 4, 7 and 8 drove fusion efficiently, whereas VAMP5 was unable to mediate fusion with the t-SNAREs. By expressing VAMPs 1, 3, 4, 7 and 8 at the same level, we further compared their membrane fusion activities. VAMPs 1 and 3 had comparable and the highest fusion activities, whereas VAMPs 4, 7 and 8 exhibited 30–50% lower fusion activities. Moreover, we determined the dependence of cell fusion activity on VAMP1 expression level. Analysis of the dependence data suggested that there was no cooperativity of VAMP proteins in the cell fusion reaction.

**Conclusions/Significance:**

These data indicate that VAMPs have differential membrane fusion capacities, and imply that with the exception of VAMP5, VAMPs are essentially redundant in mediating fusion with plasma membrane t-SNAREs.

## Introduction

Eucaryotic cells consist of membrane-bound organelles that have distinct functions. Transport of proteins and lipids among organelles relies on vesicles that are generated at donor organelles and then delivered to target organelles. The final event of the vesicular delivery process is the fusion of vesicles with the target organelles. SNARE (soluble N-ethylmaleimide-sensitive factor attachment protein receptor) proteins form the core machinery for vesicle fusion [1]–[3].

SNAREs belong to a superfamily of cytoplasmic oriented transmembrane proteins with more than 35 members in humans [4]. All SNAREs share a homologous sequence of 60–70 amino acids, the “SNARE motif” that contains eight heptad repeats ready for coiled-coil formation. When vesicles traffic to the vicinity of the target organelles, SNARE proteins on vesicles (v-SNAREs) and on target membranes (t-SNAREs) form *trans*-SNARE complexes to draw the two membranes toward each other and drive membrane fusion. Four α-helices contributed by the SNARE motifs in v- and t-SNAREs intertwine to form an extremely stable four-helix bundle that is characterized by 16 layers of mostly hydrophobic interactions between amino acid side chains [5]. Assembly of *trans*-SNARE complexes starts from the N-termini and proceeds to the C-termini in a zippering fashion [6]. Energy made available from the assembly of *trans*-SNARE complexes is used to drive the fusion of lipid bilayers [7]–[9]. After membrane fusion, the SNARE complexes become *cis*-complexes in the target membranes. The adapter protein SNAP (soluble NSF attachment protein) and the ATPase NSF (*N*-ethylmaleimide-sensitive factor) dissociate *cis*-SNARE complexes at the expense of ATP [10], [11] to free SNAREs for the next round of fusion.

The SNARE proteins that mediate synaptic exocytosis are well-characterized. In synapses, the v-SNARE vesicle-associated membrane protein 2 (VAMP2) is present in synaptic vesicles, while t-SNAREs syntaxin1 and synaptosomal-associated protein of 25 kDa (SNAP-25) are located in the plasma membrane. Before the assembly of *trans*-SNARE complexes, syntaxin1 and SNAP-25 constitute a t-SNARE acceptor complex for VAMP2 [12]. One α-helix from VAMP2, one α-helix from syntaxin1 and two α-helices from SNAP-25 form the four-helix bundle to drive the fusion of synaptic vesicles with the plasma membrane [5].

Individuals of the SNARE family localize to distinct organelles [13], suggesting that each SNARE has selective roles in vesicle trafficking events. The 7 vesicle-associated membrane proteins (VAMPs) reside in various post-Golgi vesicular compartments, and mediate vesicle fusion with the plasma membrane, the *trans*-Golgi network (TGN) and endosomes. In particular, VAMP1 (synaptobrevin 1) and VAMP2 (synaptobrevin 2) mediate regulated exocytosis in neurons and endocrine cells [14]–[16]. Enriched in recycling endosomes and endosome-derived vesicles [17], VAMP3 (cellubrevin) has been implicated in the secretion of α-granules in platelets [18], [19], the recycling of transferrin receptors to the cell surface [20], and vesicular trafficking of integrins [21], [22]. Present primarily in the TGN, VAMP4 participates in transport between the TGN and endosomes [23], [24] and in homotypic fusion of early endosomes [25]. Preferentially expressed in muscle cells, VAMP5 (myobrevin) is associated with the plasma membrane and intracellular vesicles [26]. In addition to apical exocytosis in polarized epithelial cells [27], [28], the tetanus neurotoxin-insensitive VAMP (VAMP7) is involved in vesicular transport from endosomes to lysosomes [29]. Preferentially associated with early endosomes [30], [31], VAMP8 (endobrevin) is required in regulated exocytosis in pancreatic acinar cells [32].

VAMPs 3, 4, 7 and 8 have broad tissue distribution [17], [30]. Originally identified in nervous tissues, VAMPs 1 and 2 are also detected in skeletal muscle, fat and other tissues [33]–[37]. Therefore, in mammalian cells, multiple VAMPs are present to mediate post-Golgi vesicle trafficking. To fully understand the specific role of each VAMP in vesicular transport and fusion, it is important to determine if VAMPs have differential membrane fusion activities. An ideal experimental system to answer this question will require a quantitative membrane fusion assay and equal expression of VAMP proteins. In the current study, we developed a cell fusion assay that quantifies SNARE-mediated fusion events by activated expression of β-galactosidase, and used immunostaining and flow cytometry to measure and titrate the expression levels of VAMPs. By pairing VAMPs with 2 plasma membrane t-SNARE complexes, syntaxin1/SNAP-25 [12] and syntaxin4/SNAP-25 [38], we compared their membrane fusion activities.

## Results

### An Enzymatic Cell Fusion Assay

**Figure 1:**
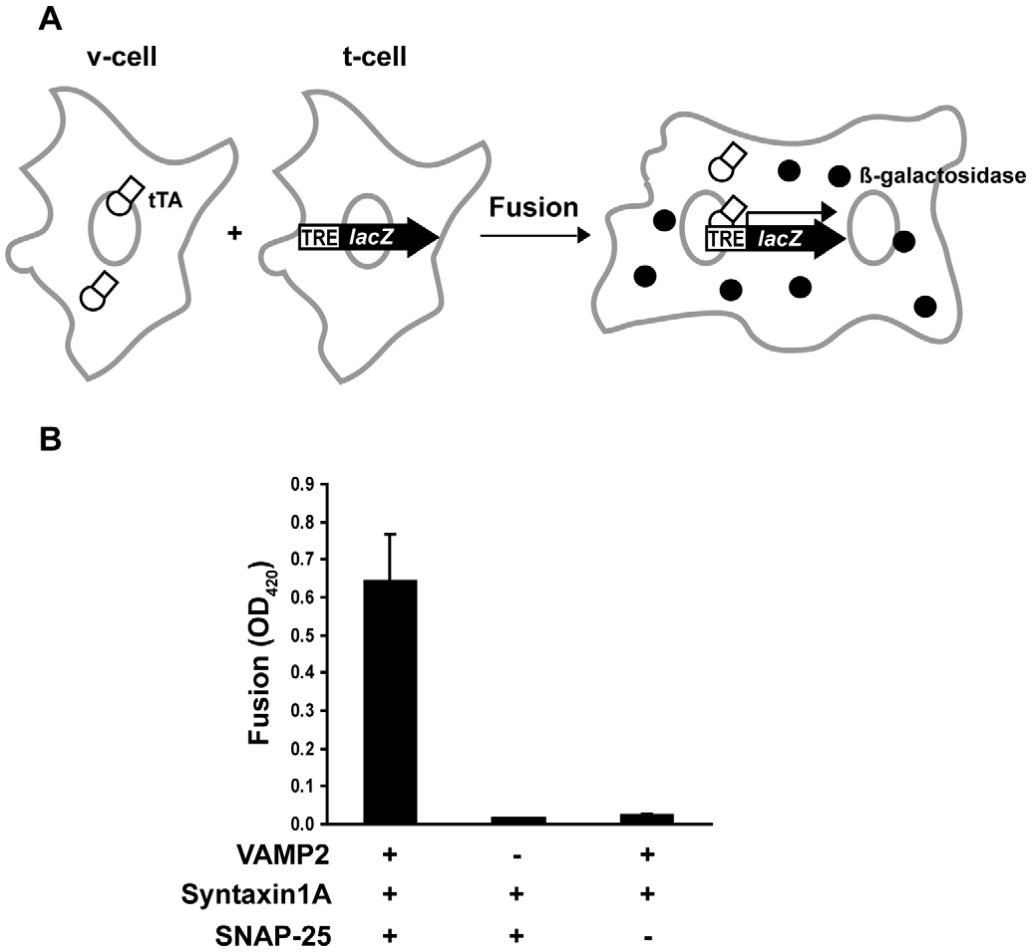
Enzymatic cell fusion assay. (A) In COS-7 cells that expressed v-SNARE proteins on the surface (v-cells), flipped v-SNARE was cotransfected with a plasmid that encodes the tetracycline-controlled transactivator (tTA). In COS-7 cells that expressed t-SNARE proteins on the surface (t-cells), flipped t-SNAREs were cotransfected with a reporter plasmid that encodes β-galactosidase under control of the tetracycline-response element (TRE-*LacZ*). 24 h after transfection, v-cells were detached from cell culture dishes, and then overlaid on t-cells. Fusion of the v- and t-cells led to the binding of tTA to TRE and expression of β-galactosidase. (B) Cell fusion depends on the interactions of v- and t-SNAREs. v-cells that expressed tTA and VAMP2 were incubated with t-cells that expressed TRE-*LacZ*, syntaxin1 and SNAP-25. After 24 h, the cells were lysed and the activity of β-galactosidase in the lysates was determined using a colorimetric method by absorbance at 420 nm. Only baseline β-galactosidase activity was detected when either flipped VAMP2 or SNAP-25 was omitted from transfection. Error bars represent standard deviation of four independent experiments.

In previous studies [9], [39], we showed that “flipped” SNAREs ectopically expressed at the cell surface drive cell-cell fusion, demonstrating that SNAREs are sufficient to fuse cellular membranes and providing a reconstituted system to study SNARE-mediated membrane fusion. Nevertheless, because the cell fusion assay is based on microscopic analysis, it becomes less efficient when used to analyze multiple v-/t-SNARE combinations quantitatively. To develop a more quantitative cell fusion assay, we took advantage of the strong transcriptional activation by binding of the tetracycline-controlled transactivator (tTA) to the tetracycline-response element (TRE) [40]. To this end, two plasmids in CLONTECH's Tet-Off gene expression system were used. The first plasmid pTet-Off encodes the transcriptional activator tTA, and the second plasmid pBI-G encodes the *LacZ* gene under control of the tetracycline-response element (TRE-*LacZ*). In the absence of tTA, transcription of the *LacZ* gene in TRE-*LacZ* is silent. When tTA is present, it binds to the TRE and activates the transcription of *LacZ*, resulting in the expression of β-galactosidase. We reasoned that if tTA was located in the cells that expressed flipped v-SNARE proteins on the cell surface (v-cells) and TRE-*LacZ* was located separately in the cells that expressed flipped t-SNARE proteins on the cell surface (t-cells), β-galactosidase would not be expressed. Fusion of the v- and t-cells would result in the binding of tTA to TRE and the transcriptional activation of *LacZ* (Fig. 1A). Since more cell fusion events lead to the binding of more tTA molecules to TRE and thus increased transcription of *LacZ*, the level of β-galactosidase expression is expected to be proportional to the number of cell fusion events.

To examine feasibility of the assay, we used the well-characterized neuronal SNAREs (v-SNARE VAMP2, and t-SNAREs syntaxin1 and SNAP-25), which drive cell fusion when expressed on the cell surface [9]. VAMP2 was coexpressed with tTA in v-cells, and syntaxin1 and SNAP-25 were coexpressed with TRE-*LacZ* in t-cells. When the v- and t-cells were combined, robust β-galactosidase expression was detected by a colorimetric method within 24 h (Fig. 1B). However, when either VAMP2 was not expressed in the v-cells or SNAP-25 was not expressed in the t-cells, only small baseline β-galactosidase activity was detected (Fig. 1B), indicating that cell fusion and expression of β-galactosidase relied on interactions of the v- and t-SNAREs. These experiments demonstrated that the enzymatic cell fusion assay identifies fusogenic pairings between v- and t-SNAREs efficiently. The baseline β-galactosidase expression was probably caused by background transcription of TRE-*LacZ* in the absence of tTA binding or by spreading of the reporter plasmids among the v- and t-cells that did not involve cell fusion.

### Fusogenic Pairings of VAMPs and plasma membrane t-SNAREs

The enzymatic cell fusion assay was used to investigate if all 7 VAMPs form fusogenic pairings with the plasma membrane t-SNAREs syntaxin1/SNAP-25 and syntaxin4/SNAP-25. The flipped VAMP2, VAMP3, syntaxin1, syntaxin4 and SNAP-25 constructs have been reported [9], [39]. Since the current focus is membrane fusion capacity of v-/t-SNARE interactions but not regulation of SNARE function, we used the syntaxin1 and syntaxin4 constructs in which the inhibitory N-terminal domains of syntaxins were removed. The truncated syntaxin proteins have higher membrane fusion activities than the full-length proteins [39], [41].

**Figure 2:**
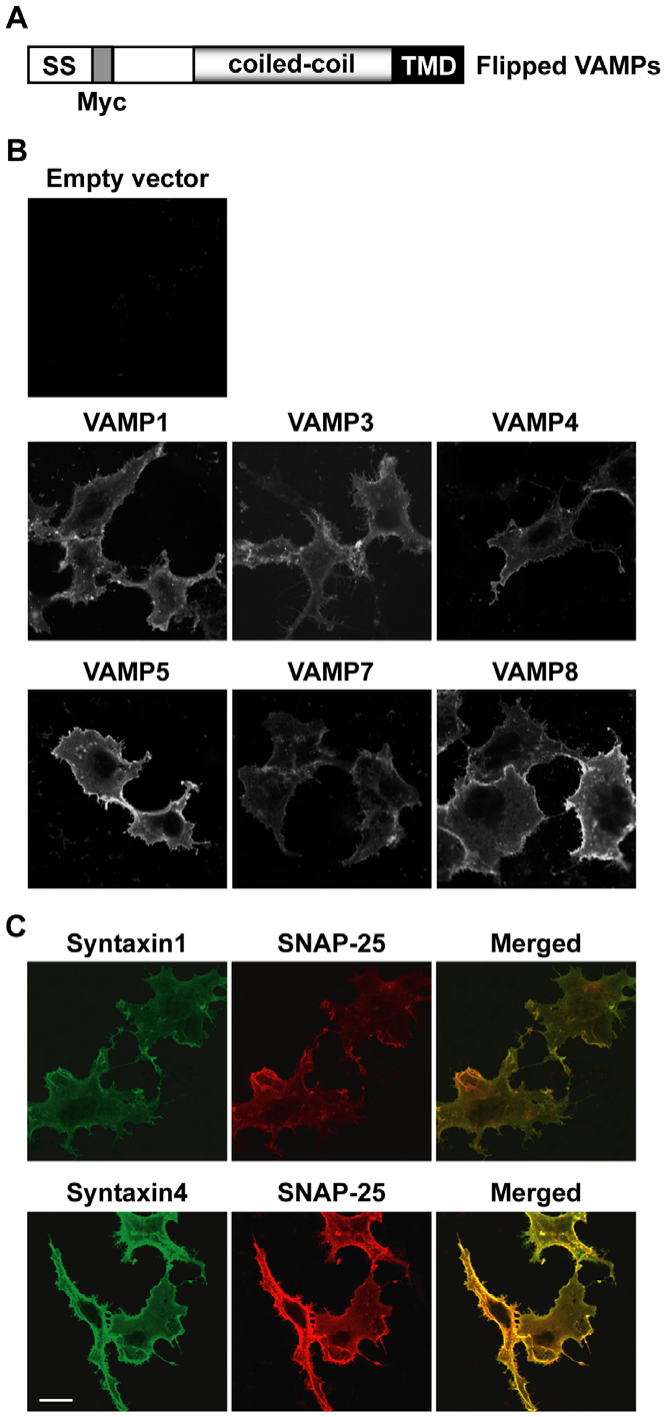
Expression of flipped SNARE proteins at the cell surface. (A) Domain structure of flipped VAMPs. The preprolactin signal sequence (SS) was fused to the N-termini of VAMPs 1, 3, 4, 5, 7 and 8. A Myc tag was inserted between the signal sequence and the VAMP proteins. (B) COS-7 cells were cotransfected with tTA and the empty vector pcDNA3.1(+), flipped VAMPs 1, 3, 4, 5, 7 or 8. 24 h after transfection, unpermeabilized cells were stained with an anti-Myc monoclonal antibody to detect the expression of VAMPs at the cell surface. (C) COS-7 cells were cotransfected with TRE-*LacZ*, flipped SNAP-25 and syntaxins 1 or 4. 24 h after transfection, unpermeabilized cells were dual labeled with antibodies. Syntaxins 1 and 4 were labeled with the anti-Myc monoclonal antibody (green), and SNAP-25 was labeled with a polyclonal antibody (red). Representative confocal microscopy images of four independent experiments are shown. Scale bar, 20 µm.

To develop constructs of flipped VAMPs 1, 4, 5, 7 and 8, the preprolactin signal sequence was fused to the N-termini of the VAMPs, and a Myc tag was inserted between the signal sequence and the N-termini (Fig. 2 A). Staining of transfected COS-7 cells with an anti-Myc antibody showed that VAMPs 1, 3, 4, 5, 7 and 8 were expressed at the cell surface (Fig. 2B). The expression of VAMPs 5 and 8 was visibly higher than VAMPs 1, 3, 4 and 7. Cell surface expression of flipped VAMP2 protein, which does not contain a Myc tag, has been described [9]. Because there are putative N-glycosylation motifs (Asn-X-Ser/Thr) in VAMPs 1, 4, 5, 7 and 8, tunicamycin (6.7 µg/ml) was included in cell culture medium to prevent N-glycosylation of these VAMP proteins. Likewise, when COS-7 cells were cotransfected with flipped syntaxin1 and SNAP-25, both t-SNARE proteins were expressed at the cell surface (Fig. 2C). When cells were cotransfected with the same amount of flipped syntaxin4 and SNAP-25, more syntaxin4/SNAP-25 proteins were detected at the cell surface than syntaxin1/SNAP-25 proteins (compare top and bottom rows in Fig. 2C). As shown previously [9], [39], SNAP-25, which does not contain a transmembrane domain, was anchored to the cell surface by forming complexes with syntaxins.

**Figure 3:**
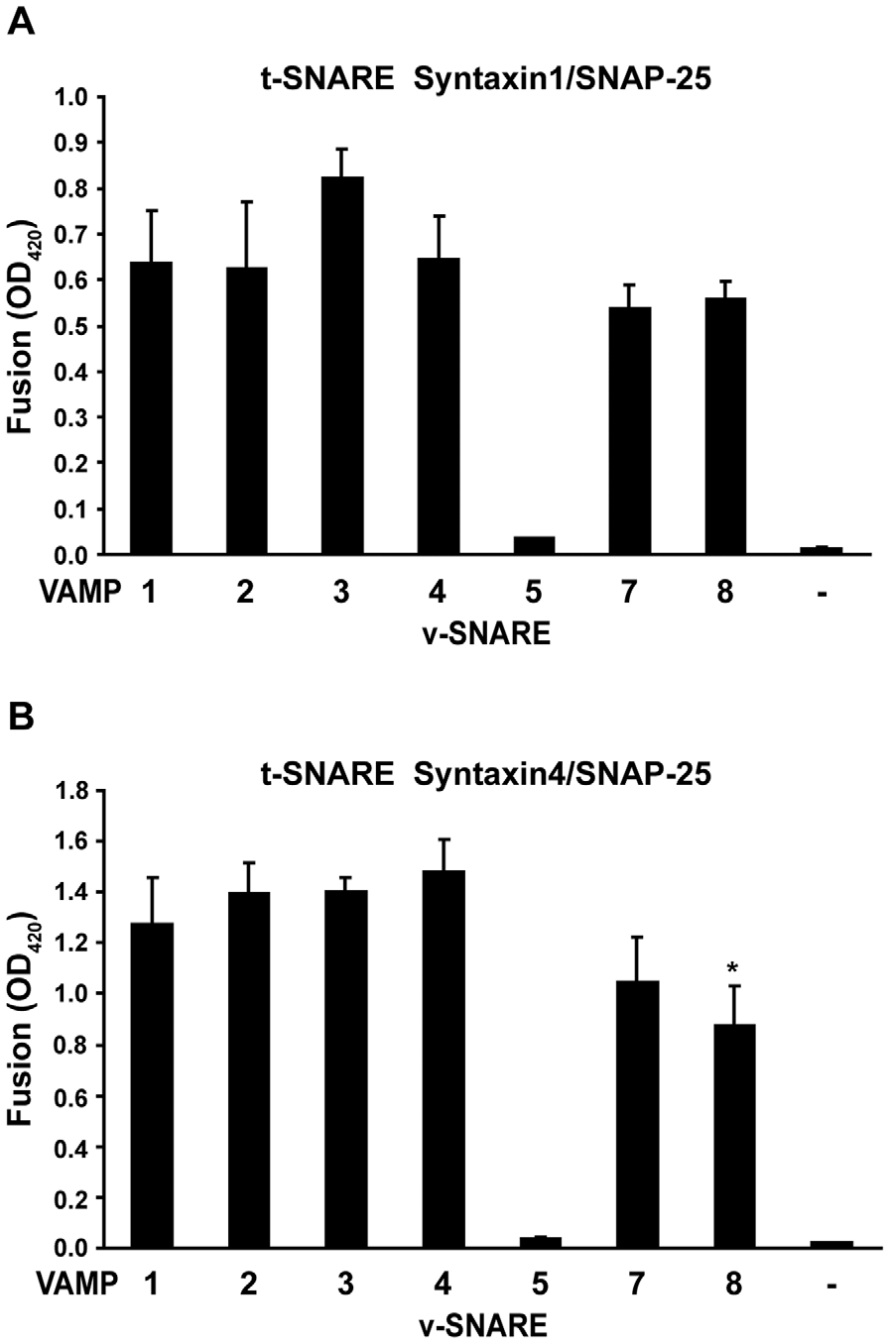
Cell fusion by VAMPs and plasma membrane t-SNAREs. 24 h after transfection, v-cells that expressed tTA and VAMPs 1, 2, 3, 4, 5, 7 or 8 were combined with t-cells that expressed TRE-*LacZ* and (A) syntaxin1/SNAP-25 or (B) syntaxin4/SNAP-25. After 24 h, cell fusion was quantified using the enzymatic cell fusion assay. Control cells (-VAMP) were cotransfected with the empty vector and the plasmid encoding tTA. Only baseline β-galactosidase activity was detected when the control cells were incubated with the t-cells. The flipped SNARE plasmids were transfected at the same concentration. Error bars represent standard deviation of three independent experiments. * *P*<0.05 vs. VAMP1.

Using the enzymatic fusion assay (Fig. 1), we examined the fusogenic pairings between the VAMPs and t-SNAREs. Robust β-galactosidase expression was detected when the v-cells expressing VAMPs 1, 2, 3, 4, 7 or 8 were combined with the t-cells expressing syntaxin1/SNAP-25 (Fig. 3A) or syntaxin4/SNAP-25 (Fig. 3B), indicating that these VAMPs mediated membrane fusion with plasma membrane t-SNAREs. With syntaxin1/SNAP-25, the 6 VAMPs drove fusion to a similar degree. With syntaxin4/SNAP-25, VAMP8 fused less efficiently than VAMPs 1, 2, 3 and 4 (31% lower fusion activity and *P* = 0.046 vs. VAMP1, Fig. 3B). In contrast, when the v-cells expressing VAMP5 were combined with the t-cells, we detected only baseline β-galactosidase activity, which was comparable to the β-galactosidase activity produced by the control cells that did not express v-SNAREs (Figs. 3A and B), suggesting that VAMP5 did not drive membrane fusion with syntaxin1/SNAP-25 or syntaxin4/SNAP-25. The stronger fusion activities of syntaxin4/SNAP-25 than syntaxin1/SNAP-25 (compare Figs. 3A and B) can be explained by higher cell surface expression of syntaxin4/SNAP-25 (Fig. 2C) and higher fusion activity of syntaxin4 than syntaxin1 (see Fig. 5 below). Taken together, the data shown in Fig. 3 indicated that VAMPs 1, 2, 3, 4, 7 and 8, but not VAMP5, drove membrane fusion when partnering with plasma membrane t-SNAREs.

### Comparison of membrane fusion activities of VAMPs

**Figure 4:**
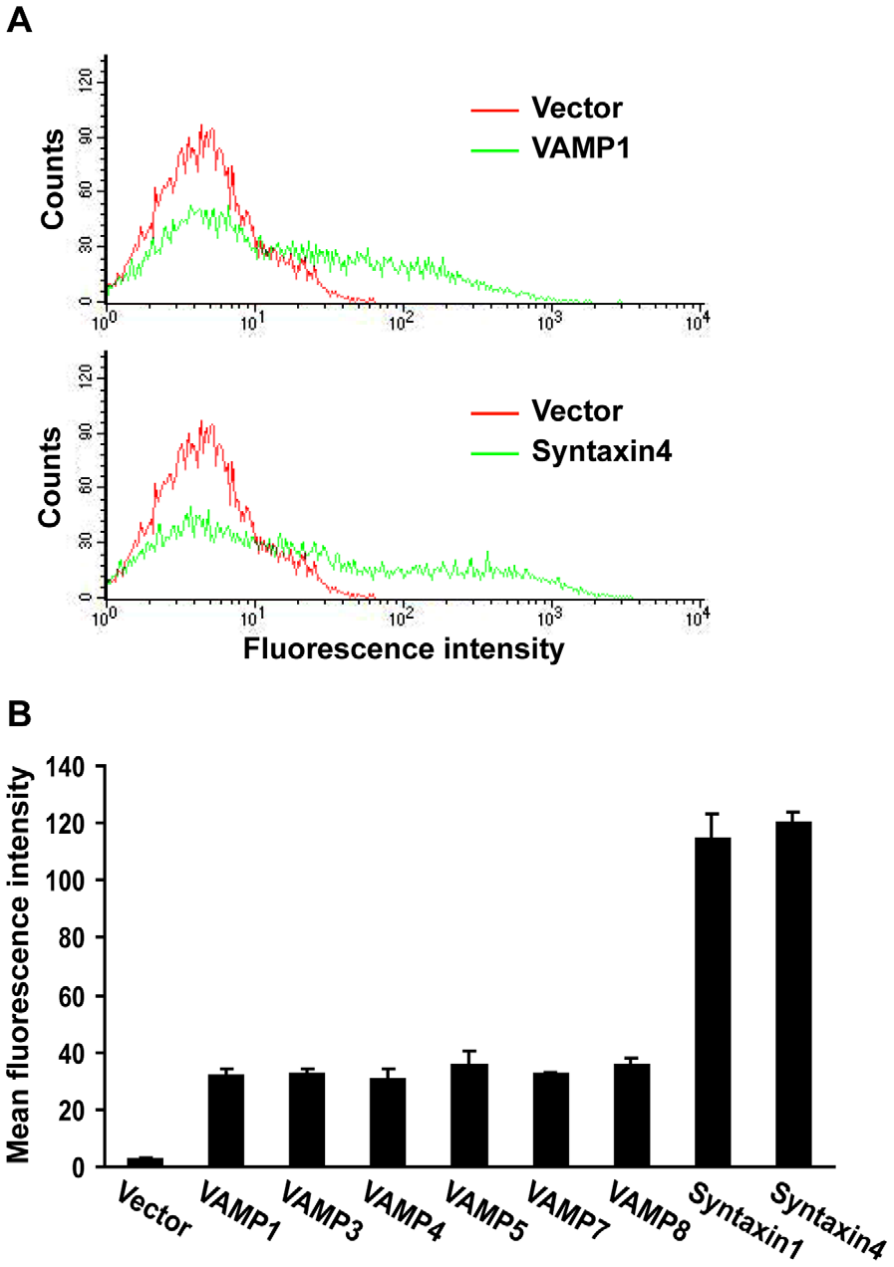
FACS analysis of SNARE expression at the cell surface. 24 h after cotransfection with tTA and the empty vector, flipped VAMPs 1, 3, 4, 5, 7 or 8 (v-cells), or 24 h after cotransfection with TRE-*LacZ*, flipped SNAP-25 and syntaxins 1 or 4 (t-cells), unpermeabilized COS-7 cells were stained with an anti-Myc antibody, and then analyzed by flow cytometry. (A) Representative FACS profiles of the cells transfected with the empty vector, flipped VAMP1 or syntaxin4/SNAP-25. (B) To express the v- and t-SNAREs at same level at the cell surface, flipped SNARE plasmids were transfected at the following concentrations (per 10 cm^2^ growth area, *i.e.*, per well in 6-well plates): VAMP1, 0.2 µg; VAMP3, 0.5 µg; VAMP4, 0.5 µg; VAMP5, 0.05 µg; VAMP7, 1.0 µg; VAMP8, 0.1 µg; syntaxin1, 0.5 µg; syntaxin4, 0.05 µg. tTA, TRE-*LacZ* and flipped SNAP-25 were cotransfected at 1 µg per 10 cm^2^ growth area. The mean fluorescence intensity of staining of the SNAREs was determined by FACS analysis. Error bars represent standard deviation of four independent experiments.

In order to compare the membrane fusion capacities of VAMPs, the v-SNAREs need to be expressed at the same level. Since flipped VAMPs 1, 3, 4, 5, 7 and 8, and syntaxins 1 and 4 contain a Myc tag, we measured cell surface expression of the SNARE proteins by anti-Myc staining and flow cytometry (Fig. 4A). When COS-7 cells were transfected with the flipped SNARE plasmids at the same concentration, cell surface expression of VAMPs 5 and 8 was more than 2 fold higher than VAMPs 1, 3, 4 and 7, and cell surface expression of syntaxin4 was 1.8 fold higher than syntaxin1 (Fig. S1). To express the v- and t-SNAREs at the same level, we titrated and optimized the concentration of each flipped SNARE plasmid used in transfection. Under such conditions, VAMPs 1, 3, 4, 5, 7 and 8 were expressed at same level at the cell surface, while syntaxins 1 and 4 were expressed at the same level (Fig. 4B). Because the flipped VAMP2 protein does not contain a Myc tag [9], we were not able to compare its expression with the other VAMPs.

**Figure 5:**
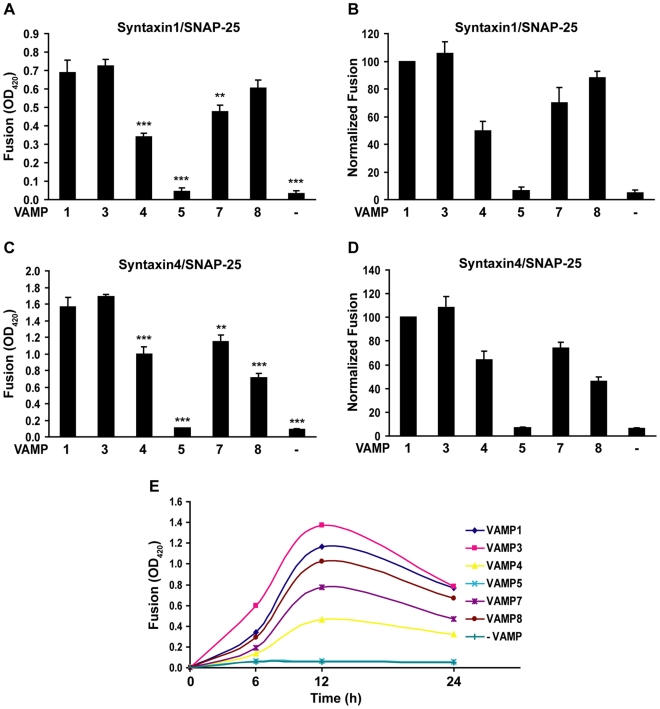
Comparison of fusion activities of VAMPs. To express VAMPs 1, 3, 4, 5, 7 and 8 at same level at the cell surface, and to express syntaxin1/SNAP-25 and syntaxin4/SNAP-25 at the same level, plasmids were transfected at the concentrations described in the legend of Fig. 4B. (A and C) 24 h after mixing the v- and t-cells, cell fusion was quantified using the enzymatic fusion assay. Error bars represent standard deviation of four independent experiments. **P<0.01 vs. VAMP1; *** *P*<0.001 vs. VAMP1. (B and D) The fusion activities (OD_420_) of the control cells expressing the empty vector (-VAMP) and the v-cells expressing VAMPs 3, 4, 5, 7 or 8 were normalized to the fusion activity of the v-cells expressing VAMP1. Error bars represent standard deviation of the four independent experiments. (E) Time course of cell fusion. 6, 12 or 24 h after combining the v-cells expressing different VAMPs with the t-cells expressing syntaxin1/SNAP-25, cell fusion was quantified. The time-course curve of the VAMP5-expressing cells overlapped completely with the time-course curve of the control cells. Shown is a representative of two independent experiments.

After expressing VAMPs 1, 3, 4, 5, 7 and 8 at the same level, we compared their membrane fusion activities using the enzymatic cell fusion assay. With syntaxin1/SNAP-25, VAMPs 1, 3, and 8 had comparable and the highest fusion activities, whereas VAMPs 4 and 7 had 50% and 30% lower fusion activities, respectively (Figs. 5A and B). With syntaxin4/SNAP-25, VAMPs 1 and 3 had comparable and the highest fusion activities, whereas VAMPs 4, 7 and 8 had 36%, 26% and 54% lower fusion activities, respectively (Figs. 5C and D). As expected, only baseline β-galactosidase activity was detected when VAMP5 was paired with the t-SNAREs. These data indicated that VAMPs have differential membrane fusion activities with plasma membrane t-SNAREs. When expressed at the same level, syntaxin4 drove fusion more efficiently than syntaxin1 (compare Figs. 5A and C), suggesting that syntaxin4 has higher membrane fusion activity than syntaxin1.

Having shown that VAMPs have differential fusion activities, we sought to determine if membrane fusion by VAMPs follows different time courses. Cell fusion by VAMPs 1, 3, 4, 5, 7 or 8 and syntaxin1/SNAP-25 was measured at 6, 12 or 24 h after combining the v- and t-cells. The results in Fig. 5E showed that cell fusion by VAMPs 1, 3, 4, 7 and 8 followed similar time courses, with peak β-galactosidase activity detected at 12 h. The slight drop of β-galactosidase activities at 24 h was likely caused by detachment of some of the fused cells from cell culture plates.

**Figure 6:**
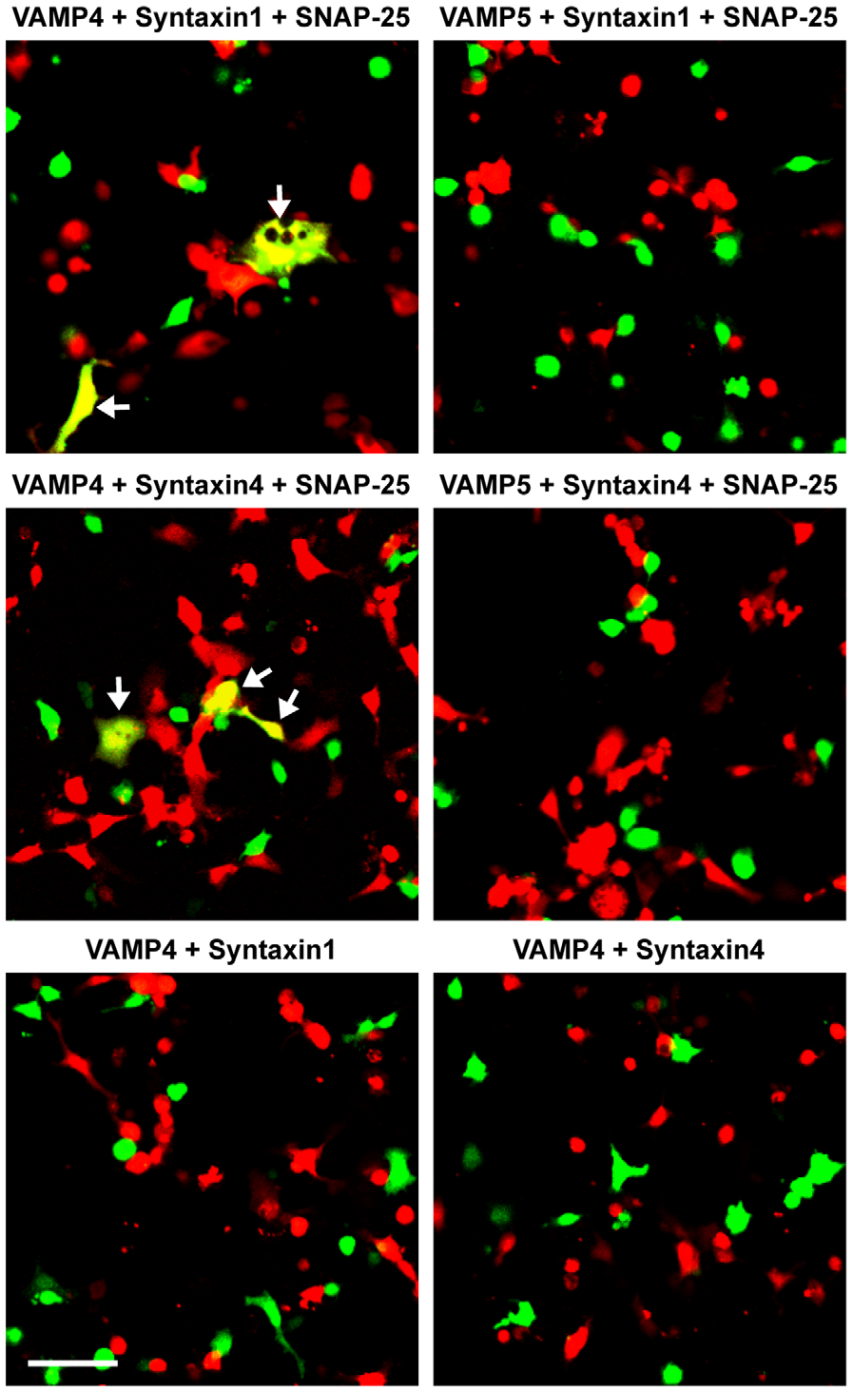
VAMP5 does not mediate membrane fusion with plasma membrane t-SNAREs. v-cells expressing VAMPs 4 or 5 were labeled by the green fluorescent protein EGFP. t-cells expressing syntaxin1/SNAP-25, syntaxin4/SNAP-25, syntaxin1 alone, or syntaxin4 alone were labeled by the red fluorescent protein DsRed2. The v- and t-cells were combined for 24 h. EGFP and DeRed2 were imaged sequentially on a confocal microscope before merging the images. Representative confocal images of three independent experiments are shown. Arrows indicate fused cells with a yellow cytoplasm. Scale bar, 100 µm.

To rule out the possibility that the baseline β-galactosidase activity detected in the VAMP5 combinations (Fig. 5) was caused by residual fusion activity of VAMP5, we performed the microscopic cell fusion assay [39], which analyzes individual cell fusion events and can detect rare fusion events. In this assay, flipped v-SNAREs are coexpressed with the green fluorescent protein EGFP in v-cells and flipped t-SNAREs are coexpressed with the red fluorescent protein DsRed2 in t-cells. Fusion of the v- and t-cells results in fused cells whose cytoplasm is yellow under fluorescence microscope (Fig. 6). As predicted by the enzymatic cell fusion results, VAMP4 drove cell fusion with syntaxin1/SNAP-25 and syntaxin4/SNAP-25 in the microscopic assay (Fig. 6). In multiple experiments, no cell fusion was observed using the microscopic assay when v-cells expressing VAMP5 were combined with t-cells expressing syntaxin1/SNAP-25 or syntaxin4/SNAP-25 (Fig. 6). Based on the results using both the enzymatic and microscopic cell fusion assays, we concluded that VAMP5 is unable to mediate membrane fusion with the plasma membrane t-SNAREs.

### Dependence of cell fusion activity on cell surface density of VAMP1

**Figure 7:**
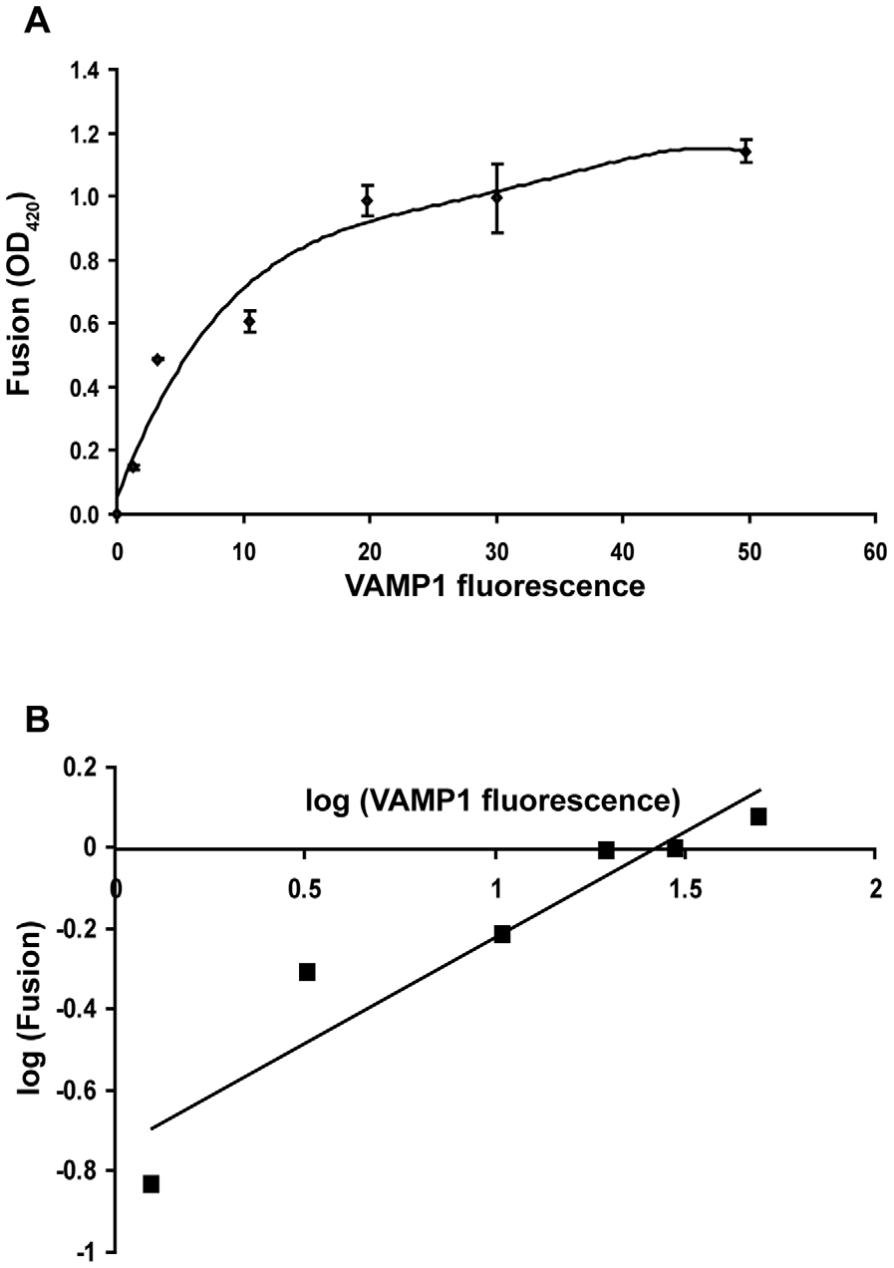
Dependence of cell fusion activity on cell surface density of VAMP1. (A) v-cells were cotransfected with tTA (1 µg per 10 cm^2^ growth area) and increasing amount of the flipped VAMP1 plasmid (per 10 cm^2^ growth area): 0.01, 0.02, 0.05, 0.1, 0.2 and 0.5 µg. After the v-cells were combined with the t-cells expressing syntaxin1/SNAP-25 for 24 h, cell fusion was quantified. In parallel experiments, the expression level of VAMP1 proteins at surface of the v-cells was determined by staining with the anti-Myc antibody and FACS analysis. A polynomial regression curve was generated to model the relationship between cell fusion activity (OD_420_) and mean fluorescence intensity of VAMP1 staining. Error bars represent standard deviation of three independent experiments. (B) Log-log plot of cell fusion activity vs. mean fluorescence intensity of VAMP1 staining. The solid line shows a linear regression, which yields a slope of 0.52 with a correlation coefficient of 0.901.

We next asked how many SNARE complexes cooperate to drive the cell fusion reaction. To this end, we determined the dependence of cell fusion activity on cell surface expression level of VAMP1, which has high fusion activity (Fig. 5). COS-7 cells were transfected with increasing concentrations of the flipped VAMP1 plasmid. At each concentration, we measured the cell surface expression level of VAMP1 proteins using immunostaining and flow cytometry, and determined cell fusion activity of VAMP1 with syntaxin1/SNAP-25 using the enzymatic fusion assay. Cell fusion activity was then plotted as a function of the mean fluorescence intensity of VAMP1 staining (Fig. 7A). The correlation was best fit with a polynomial regression. The hyperbolic instead of sigmoidal correlation (Fig. 7A) suggests that there was no cooperativity of VAMP1 proteins in driving cell fusion.

The log-log plot was used to determine the cooperativity of the viral fusion protein hemagglutinin in membrane fusion [42]. Using log-log plot, we further analyze the cooperativity of VAMP1 proteins. If three VAMP1 (V) proteins are required in the cell fusion reaction, *i.e.*, V+V+V → Fusion, the rate of fusion (F)  = *k* [V]^3^. Therefore, log (F)  = log (*k*) +3 log [V], and the slope of the resulting log-log plot will be 3. A log-log analysis of the dependence of cell fusion activity on VAMP1 cell surface density is shown in Fig. 7B. In this analysis, F =  fusion activity (OD_420_) and [V]  =  mean fluorescence intensity of VAMP1 staining. Linear regression was performed to model the log-log correlation (Fig. 7B), and the resulting slope was 0.52. When only the 3 data points with the lowest VAMP1 expression were modeled by linear regression, the slope was 0.62. These analyses further suggest that the cell fusion reaction did not involve concerted action of VAMP1 proteins.

## Discussion

In this study, we have developed a cell fusion assay that quantifies SNARE-mediated fusion events by activated expression of β-galactosidase. Using this assay, we showed that VAMPs 1, 2, 3, 4, 7 and 8 mediated fusion efficiently with syntaxin1/SNAP-25 and syntaxin4/SNAP-25, whereas VAMP5 did not drive fusion with the t-SNAREs. Using immunostaining and flow cytometry as a measurement of cell surface SNARE expression, we expressed VAMPs 1, 3, 4, 7 and 8 at the same level and further compared their membrane fusion activities. With syntaxin1/SNAP-25, VAMPs 1, 3 and 8 had comparable and the highest fusion activities, while VAMPs 4 and 7 had 50% and 30% lower fusion activities, respectively. With syntaxin4/SNAP-25, VAMPs 1 and 3 had comparable and the highest fusion activities, while VAMPs 4, 7 and 8 had 36%, 26% and 54% lower fusion activities, respectively. Taken together, these data indicate that VAMPs have differential membrane fusion activities. However, when expressed at higher levels, VAMP4 drove membrane fusion as efficiently as VAMPs 1 and 3 (Fig. 3). In addition, the differences of fusion activities among the fusogenic VAMPs are within a factor of 2, implying that with the exception of VAMP5, VAMPs are essentially redundant in mediating membrane fusion with plasma membrane t-SNAREs.

VAMPs 1, 2, 3, 7 and 8 are known to mediate vesicle fusion with the plasma membrane. The current study provides additional evidence that vesicles that carry either one of these 5 VAMPs are capable of fusing with the plasma membrane. Since these VAMPs are functionally redundant, they can compensate each other in loss of function studies [43]. VAMP4 mainly localizes to the TGN [23], [44], with a small percentage (∼6%) of VAMP4 proteins present in endosomal vesicles [23]. Forming a v-/t-SNARE complex with syntaxin16, syntaxin6 and vti1a, VAMP4 is involved in vesicle trafficking from endosomes to the TGN [24]. Interacting with syntaxin13, syntaxin6 and vti1a, VAMP4 mediates homotypic fusion of early endosomes [25]. The current data show that VAMP4 drives membrane fusion with plasma membrane t-SNAREs, suggesting that VAMP4 may mediate a third vesicle fusion event - fusion of vesicles with the plasma membrane.

VAMP5 is preferentially expressed in the skeletal muscle and heart and its expression increases during myogenesis [26]. In muscle cells, VAMP5 is mainly associated with the plasma membrane as well as intracellular vesicles [26], [45]. Our data show that VAMP5 is unable to mediate membrane fusion with plasma membrane t-SNAREs, suggesting that VAMP5 does not mediate vesicle fusion with the plasma membrane. The function of VAMP5 as a v-SNARE warrants further investigation.

Many kinds of secretory molecules and transmembrane proteins are delivered to the cell surface by exocytosis. If VAMPs are functionally redundant, what roles do SNAREs play in achieving specificity of vesicle fusion at the plasma membrane? First, VAMPs have distinct subcellular localizations (for example, VAMP8 is mainly associated with early endosomes, whereas VAMP3 is enriched in recycling endosomes). Specific VAMP thus mediates the fusion of different populations of vesicles with the plasma membrane. Second, syntaxins are present in distinct domains at the plasma membrane. In polarized epithelial cells, syntaxin4 is restricted to the basolateral domain of plasma membrane [46], whereas syntaxin1 is delivered to both the apical and basolateral domains [47]. Therefore, VAMPs may partner with syntaxin4 to deliver cargo molecules to the basolateral side of the plasma membrane, and partner with syntaxin1 to deliver cargo molecules to both the apical and basolateral sides. Furthermore, specificity of vesicle fusion is enhanced by the binding of regulatory proteins such as Munc18 to SNAREs [48].

The number of SNARE complexes that cooperate to mediate vesicle fusion is under active investigation. Using various model systems including titration of SNARE inhibitors and liposome fusion assays, 1 to 11 SNARE complexes are estimated to be needed for membrane fusion [49]–[52]. In intact neuroendocrine cells, the fast phase of exocytosis requires at least 3 SNARE complexes, while the slower phase of exocytosis may occur with 1 SNARE complex [53]. In this study, we determined the dependence of cell fusion activity on cell surface density of VAMP1, and did not observe cooperativity of VAMP1 proteins in the cell fusion reaction. These data suggest that concerted action of multiple SNARE complexes is not required to fuse cellular membranes. However, to achieve fast exocytosis in intact cells, concerted action of multiple SNARE complexes is clearly needed. Such cooperativity of SNARE complexes may be organized by the binding of regulatory proteins such as Munc18 and synaptotagmins.

The original cell fusion assay [9] analyzes SNARE-mediated fusion events by fluorescence microscopy. The enzymatic cell fusion assay described here utilizes controlled expression of β-galactosidase and spectrometric measurement, and thus dramatically simplify the quantification of fusion events. In addition, flow cytometry is now used to measure the levels of SNARE expression. With these modifications, the cell fusion assay offers a quantitative approach for examining the fusogenic pairings of v- and t-SNAREs and for high-throughput studies.

## Materials and Methods

### Cell culture and reagents

COS-7 cells were obtained from the American Type Culture Collection, and cultured in Dulbecco Modified Eagle's Medium (DMEM) supplemented with 4.5 g/l glucose and 10% fetal bovine serum (FBS). The anti-Myc monoclonal antibody 9E10, developed by Dr. Bishop, was obtained from the Developmental Studies Hybridoma Bank maintained by the University of Iowa.

### Constructs of Flipped VAMPs

To generate flipped VAMP1, the flipped syntaxin1 plasmid (pCH9) [9] was digested with XbaI and ApaI to excise the coding region of syntaxin1. The vector fragment was purified from an agarose gel. The coding sequence of mouse VAMP1 was amplified by PCR from a mouse skeletal muscle Marathon-Ready cDNA library (CLONTECH) with primers VP1F (TCTGTCTAGATCTGCTCCAGCTCAACCGC) and VP1R (AAACGGGCCCTCAAGTAAAAAAGTAGATTACAATCACTACCACG). Using the PCR product as template, the coding sequence of VAMP1 (a.a. 2–118) was further amplified by PCR with primers CHU7F (TCTGTCTAGATCTGCTCCAGCTCAACCG) and CHU7R (TAAACGGGCCCTCAAGTAAAAAAGTAGATTACAATCACTACCAC). The new PCR product was digested with XbaI and ApaI and cloned into the XbaI and ApaI sites of pCH9, resulting in plasmid flipped VAMP1 (pCHU7).

To generate flipped VAMP4, the coding sequence of human VAMP4 (a.a. 1–141) was amplified by RT-PCR using total RNA isolated from human umbilical cord endothelial (HUVEC) cells with primers XbaVMP4 (CTGTCTAGAATGCCTCCCAAGTTTAAGCGCCACC) and VMP4Apa (AAACGGGCCCTCAAGTACGGTATTTCATGACTATAAG). The PCR product was digested with XbaI and ApaI and cloned into the XbaI and ApaI sites of pCH9, resulting in plasmid flipped VAMP4 (pCHL22).

To generate flipped VAMP5, the coding sequence of human VAMP5 was amplified by RT-PCR using the total RNA isolated from HUVEC cells with primers hVAMP5F (GCAGGAATAGAGTTGGAGCGGTG) and hVAMP5R (TCAGTTCCCAGGCCCTGAGG). Using the PCR product as template, the coding sequence of VAMP5 (a.a. 2–116) was further amplified by PCR with primers CHL20F (TCTGTCTAGAGCAGGAATAGAGTTGGAGCGG) and CHL20R (AAACGGGCCCTCAGTTCCCAGGCCCTGAG). The new PCR product was digested with XbaI and ApaI and cloned into the XbaI and ApaI sites of pCH9, resulting in plasmid flipped VAMP5 (pCHL20).

To generate flipped VAMP7, the coding sequence of mouse VAMP7 was amplified by PCR from the skeletal muscle Marathon-Ready cDNA library with primers mVAMP7F (ATGGCCATTCTTTTTGCTGTTGTTG) and mVAMP7R (TTATTTCTTCACACAGTTTGGCCATG). Using the PCR product as template, the coding sequence of VAMP7 (a.a. 2–220) was further amplified by PCR with primers CHU31F (TCTGTCTAGAGCCATTCTTTTTGCTGTTGTTGC) and CHU31R (AAACGGGCCCTTATTTCTTCACACAGTTTGGCCATG). The new PCR product was digested with XbaI and ApaI and cloned into the XbaI and ApaI sites of pCH9, resulting in plasmid flipped VAMP7 (pCHU31).

To generate flipped VAMP8, the coding sequence of mouse VAMP8 was amplified by PCR from the skeletal muscle Marathon-Ready cDNA library with primers mVAMP8F (ATGGAGGAGGCCAGTGGGAG) and mVAMP8R (TTAAGTGGGGATGGTACCAGTGGC). Using the PCR product as template, the coding sequence of VAMP8 (a.a. 2–101) was further amplified by PCR with primers CHU32F (TCTGTCTAGAGAGGAGGCCAGTGGGAGTG) and CHU32R (AAACGGGCCCTTAAGTGGGGATGGTACCAGTGG). The new PCR product was digested with XbaI and ApaI and cloned into the XbaI and ApaI sites of pCH9, resulting in plasmid flipped VAMP8 (pCHU32).

*Pfu* DNA polymerase (Stratagene) was used for PCR cloning. SuperScript III reverse transcriptase (Invitrogen) was used for reverse transcription. All coding sequences were confirmed by DNA sequencing.

### Immunostaining of SNAREs at the cell surface

The day before transfection, 3×10^4^ COS-7 cells were seeded on sterile 12-mm glass coverslips contained in 24-well plates. In the cells that expressed flipped v-SNARE proteins (v-cells), 0.25 µg of the plasmid that encodes tTA (pTet-Off, CLONTECH) was cotransfected with 0.25 µg of the flipped VAMP constructs in each well. In the cells that expressed flipped t-SNARE proteins (t-cells), 0.25 µg of the plasmid encoding TRE-LacZ (pBI-G, CLONTECH) was cotransfected with 0.25 µg each of flipped SNAP-25 and syntaxins 1 or 4 in each well. Transfection was done with Lipofectamine according to the manufacturer's instructions (Invitrogen). 24 h after transfection, the COS-7 cells were fixed with 4% paraformaldehyde in PBS++ (PBS supplemented with 0.1 g/l CaCl_2_ and 0.1 g/l MgCl_2_). Primary antibodies were incubated with the cells at the following dilutions: anti-Myc monoclonal antibody 9E10, neat hybridoma culture supernatant; and anti-SNAP-25 polyclonal antibody (Synaptic Systems), 1∶100. Fluorophore-conjugated secondary antibodies (Jackson Immunoresearch Laboratories) were used at a dilution of 1∶500. For double staining, the cells were incubated first with a mixture of the primary antibodies, and then with a mixture of the secondary antibodies. Confocal images were collected on an Olympus laser scanning confocal microscope. The images were processed with the Adobe Photoshop software.

### FACS analysis

The expression levels of SNAREs at the cell surface were measured using immunostaining and flow cytometry as described [54]. The day before transfection, 2×10^5^ COS-7 cells were seeded in each well of 6-well plates (10 cm^2^ growth area per well). 24 h after transfection with the flipped SNARE, pTet-Off and pBI-G plasmids, the cells were fixed with 1% paraformaldehyde in PBS++ for 15 min, and then blocked in 10% FBS in PBS++ for 15 min. The cells were incubated with the anti-Myc monoclonal antibody 9E10 for 60 min at room temperature. After three washes with PBS++, the cells were labeled with FITC-conjugated secondary antibodies (1∶200 dilution) for 45 min. After three washes with PBS++, the cells were scraped off the plates with a cell scraper. 15,000 cells were analyzed using a FACSCalibur flow cytometer (BD Biosciences) in the James Graham Brown Cancer Center. The mean fluorescence intensity of each sample was obtained using the CellQuest Pro software.

### Enzymatic Cell Fusion Assay

The day before transfection, 1.2×10^6^ COS-7 cells were seeded in each 100-mm tissue culture dish, and 2×10^5^ COS-7 cells were seeded in each well of 6-well plates. For v-cells, 5 µg each of flipped VAMPs 1, 2, 3, 4, 5, 7 or 8 was cotransfected with 5 µg of pTet-Off into the cells in each 100-mm culture dish. Control cells were cotransfected with empty pcDNA3.1(+) vector and pTet-Off. To prevent N-glycosylation of VAMPs 1, 4, 5, 7 and 8, v-cells expressing these VAMP proteins and control cells were incubated in cell culture medium containing 10 µg/ml of tunicamycin during transfection. Since flipped VAMP2 [9] and VAMP3 proteins [39] do not contain N-glycosylation motifs, v-cells expressing VAMPs 2 or 3 were not treated with tunicamycin. For t-cells, 1 µg each of flipped syntaxins 1 or 4, SNAP-25 and pBI-G were cotransfected into the cells in each well of the 6-well plates.

24 h after transfection, the v-cells were detached from the culture dishes with EDTA (Enzyme-free Cell Dissociation Buffer (Invitrogen)). The detached cells were counted with a hemacytometer and resuspended in HEPES-buffered DMEM supplemented with 10% FBS, 6.7 µg/ml tunicamycin and 0.67 mM DTT. Resuspended v-cells (4.8×10^5^) were added to each well already containing the t-cells. After 6, 12 or 24 h at 37°C in 5% CO_2_, the expression of β-galactosidase was measured using the β-Galactosidase Enzyme Assay System with Reporter Lysis Buffer according to the manufacturer's instructions (Promega). The cells were washed twice with PBS, and then lysed in the Reporter Lysis Buffer. Cell lysates were mixed with equal volume of Assay 2× Buffer, and the Reporter Lysis Buffer was mixed with the Assay 2× Buffer as a blank control. After 90 min, the colorimetric reaction was stopped by adding 1 M sodium carbonate. Absorbance at 420 nm was measured using a HITACHI 100-40 spectrophotometer.

To express VAMPs 1, 3, 4, 5, 7 and 8 at same level at the cell surface to compare their membrane fusion activities, the flipped VAMP and t-SNARE plasmids were transfected at different DNA concentrations, which are described in the legend of Fig. 4B. To determine the dependence of cell fusion activity on cell surface density of VAMP1, v-cells were cotransfected with 5 µg of pTet-Off and increasing amount of the flipped VAMP1 plasmid (see the legend of Fig. 7A). Polynomial regression, linear regression and log-log plot of the correlation data were performed using Microsoft Excel.

### Microscopic cell fusion assay

The microscopic cell fusion assay was performed as described [39]. The day before transfection, 1.2×10^6^ COS-7 cells were seeded in 100-mm tissue culture dishes, and 5×10^4^ COS-7 cells were seeded on sterile 12-mm glass coverslips contained in 24-well plates. To express VAMPs 4 and 5 at same level at the surface of v-cells, 2.5 µg of flipped VAMP4 or 0.25 µg of flipped VAMP5 was cotransfected with 5 µg of pEGFP-N3 into the cells grown in the 100-mm culture dishes. To express syntaxins 1 and 4 at same level at the surface of t-cells, 0.125 µg of flipped syntaxin 1 or 0.0125 µg of flipped syntaxin 4 was cotransfected with 0.25 µg each of flipped SNAP-25 and pDsRed2-N1 into the cells seeded in the 24-well plates. 24 h after transfection, the v-cells were detached and combined with the t-cells. After 24 h at 37°C in 5% CO_2_, the coverslips were gently washed once with PBS++, then fixed with 4% paraformaldehyde. Confocal images were collected on an Olympus laser scanning confocal microscope. The 488 nm argon laser line was used to excite the green fluorescent protein EGFP and the 543 nm HeNe laser line was used to excite the red fluorescent protein DsRed2. To prevent cross-contamination between EGFP and DsRed2, each channel was imaged sequentially before merging the images. Before cell fusion, the cytoplasm of v-cells showed green fluorescence whereas the cytoplasm of t-cells showed red fluorescence. Fusion of v- and t-cells resulted in fused cells whose cytoplasm was yellow in the merged channel.

## Supporting Information

Figure S1 FACS analysis of expression levels of SNAREs at the cell surface. 24 h after cotransfection with tTA and the empty vector, flipped VAMPs 1, 3, 4, 5, 7 or 8 (v-cells), or 24 h after cotransfection with TRE-LacZ, flipped SNAP-25 and syntaxins 1 or 4 (t-cells), unpermeabilized COS-7 cells were stained with an anti-Myc antibody, and then analyzed by flow cytometry. The mean fluorescence intensity of staining of the SNAREs was determined by FACS analysis. Each plasmid was transfected at 1 mg per 10 cm2 growth area. Error bars represent standard deviation of two independent experiments.
(0.01 MB PDF)
