# Supplementary figures and images for: Fusogenic Pairings of Vesicle-Associated Membrane Proteins (VAMPs) and Plasma Membrane t-SNAREs – VAMP5 as the Exception
Source: PLoS One. 2010 Dec 6;5(12):e14238. doi: 10.1371/journal.pone.0014238 (PMC2997805; doi:10.1371/journal.pone.0014238)

Figure S1

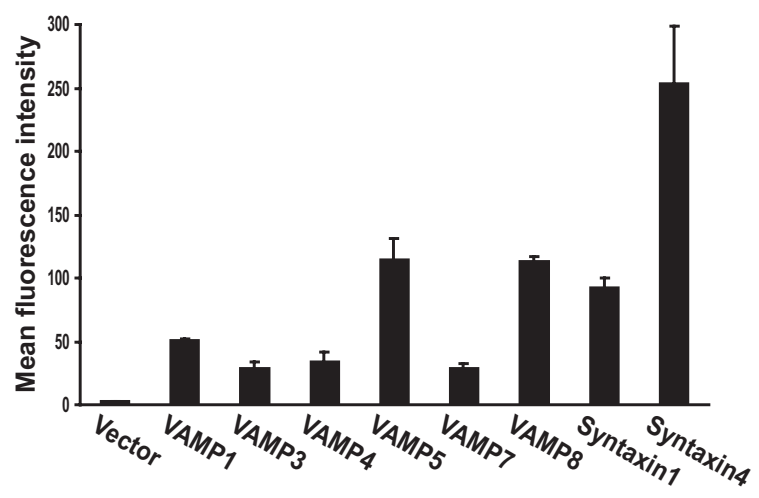

Supplement: Figure S1 — FACS analysis of expression levels of SNAREs at the cell surface. 24 h after cotransfection with tTA and the empty vector, flipped VAMPs 1, 3, 4, 5, 7 or 8 (v-cells), or 24 h after cotransfection with TRE-LacZ, flipped SNAP-25 and syntaxins 1 or 4 (t-cells), unpermeabilized COS-7 cells were stained with an anti-Myc antibody, and then analyzed by flow cytometry. The mean fluorescence intensity of staining of the SNAREs was determined by FACS analysis. Each plasmid was transfected at 1 mg per 10 cm2 growth area. Error bars represent standard deviation of two independent experiments. (0.01 MB PDF) [file pone.0014238.s001.pdf]
